# Recurrent Malignant Phyllodes Tumor of the Breast: A Case Report

**DOI:** 10.1002/ccr3.70842

**Published:** 2025-09-04

**Authors:** Ahmad Naeem Akhtar, Asim Ali, Ali Gohar, Masab Ali, Abdul Rehman, Furqan Abbas Bajwa, Muhammad Haseeb, Muhammad Husnain Ahmad

**Affiliations:** ^1^ Lahore General Hospital Lahore Punjab Pakistan; ^2^ Department of Internal Medicine Punjab Medical College Faisalabad Punjab Pakistan; ^3^ Department of Medicine Tentishev Satkynbai Memorial Asian Medical Institute Kant Kyrgyzstan

**Keywords:** excision, fibroepithelial tumors, histopathology, lumpectomy, malignant phyllodes tumor, metaplastic carcinoma of the breast, modified radical mastectomy

## Abstract

Malignant phyllodes tumors of the breast are rare fibroepithelial neoplasms with aggressive behavior and high recurrence rates. They pose significant diagnostic and therapeutic challenges due to their overlap with other malignancies, necessitating accurate diagnosis and a tailored treatment approach to improve patient outcomes. A 29‐year‐old Asian female initially underwent a lumpectomy for a right breast mass diagnosed as a phyllodes tumor on histopathology. Six months later, she developed a recurrent mass with pain, bleeding, and nipple‐areolar distortion, leading to a modified radical mastectomy. Histopathology unexpectedly revealed metaplastic carcinoma, giant cell variant. No adjuvant therapy was administered. Two years post‐mastectomy, she presented with a recurrent axillary mass associated with necrosis and purulent discharge. Imaging revealed a large necrotic soft tissue mass confined to the chest wall and axilla with subcentimeter lung nodules. No muscle invasion or intrathoracic extension was noted. Wide local excision of the axillary mass confirmed recurrent malignant phyllodes tumor. This case highlights the aggressive and recurrent nature of malignant phyllodes tumors, emphasizing their diagnostic overlap with other malignancies such as metaplastic carcinoma. Despite multiple surgical interventions, the high recurrence risk underscores the importance of precise diagnostic techniques and individualized treatment strategies.

**Summary**

- Malignant phyllodes tumors are rare, aggressive breast neoplasms.
- This case highlights diagnostic challenges from overlapping features with metaplastic carcinoma.
- Recurrence in a young woman, despite surgical interventions, underscores the tumor's aggressive nature and the importance of vigilant follow‐up and individualized management to improve outcomes in such rare presentations.

## 1 Introduction

Phyllodes tumors (PT) are rare biphasic fibroepithelial neoplasms characterized by leaf‐like growth patterns and stromal hypercellularity. It is a rare breast tumor, with a global incidence of 0.3%–1% of all breast tumors. It usually occurs in middle‐aged women (35–55 years) [1]. The incidence of PT is relatively higher in Asian women than in the Western population [2]. It is typically classified by the World Health Organization into benign, borderline, and malignant categories based on histological features such as stromal atypia, mitotic activity, and tumor margins [3, 4, 5]. Although most PTs range in size from 4 to 7 cm, a subset grows to over 10 cm and is classified as giant phyllodes tumors, which present significant treatment challenges owing to their size and aggressive behavior [1]. The standard treatment for PT is complete surgical excision with negative margins, as recommended by the National Comprehensive Cancer Network (NCCN) [6, 7]. Despite adequate surgical margins, the local relapse rate is approximately 20%, and distant metastases occur in 14%–15%, most commonly to the lungs. Lymph node metastasis is rare; enlarged nodes often result from necrosis or inflammation, and the utility of sentinel lymph node biopsy remains unclear. This case report highlights a rare and aggressive malignant giant phyllodes tumor that recurred rapidly following mastectomy and adjuvant therapy, underscoring the complexities of managing such cases. Given the diagnostic overlap and potential for rapid recurrence, this case emphasizes the critical need for precise diagnostic techniques and individualized treatment strategies to effectively manage these tumors and improve long‐term patient outcomes. This report follows the SCARE guidelines [8].

## 2 Case Presentation

A 29‐year‐old female underwent a lumpectomy for a right breast mass, which was performed at another hospital. Intraoperative findings revealed a lump in the upper quadrant of the right breast. Histopathological examination confirmed the presence of a phyllodes tumor. After 6 months, she presented to our outpatient surgery department with a painful, mobile mass in the right axilla, accompanied by bleeding and purulent, foul‐smelling discharge for 6 months. Physical examination revealed a hard, painful, fungating mass in the right axilla originating from the previous mastectomy site, with associated bleeding, purulent discharge, and skin changes, including multiple black necrotic patches. Modified radical mastectomy (MRM) was performed. Intraoperative findings revealed a 20 × 18 × 16 cm mass in the right breast. Histopathology identified it as metaplastic carcinoma, a giant cell variant. No adjuvant radiotherapy or chemotherapy was administered at that time. The patient was referred to an oncologist for further management.

Two years post‐MRM, she returned with a painful, fungating mass in the right axilla, associated with foul‐smelling purulent discharge and skin changes, including multiple black necrotic patches.

### 2.1 Differential Diagnosis, Investigation and Treatment

Radiological investigations included a computed tomography (CT) scan, which revealed a 15 × 14 cm large soft tissue mass with internal necrotic areas in the right anterolateral chest wall, involving the mastectomy bed and extending anteriorly to the axilla, reaching up to the skin. Muscle invasion or intrathoracic extension was not observed. Subcentimeter‐sized soft tissue density nodules were noted in the right middle and left lingular lobes of the lungs. Additionally, CT scans of the brain and bone showed no abnormalities. Another non‐contrast CT of the chest, abdomen, and pelvis revealed a 17 × 17.4 × 17.7 cm large soft tissue mass with internal hemorrhages and necrotic areas in the right axilla, containing internal gas locules with ulcerated skin. Right pleural effusion with soft tissue density lesions was noted in the right middle and left lingular lobes. High‐resolution CT (HRCT) of the chest revealed mild right‐sided effusion, subpleural nodules, irregular septal thickening, and thickening of the bronchovascular bundle, mainly in the right upper and middle lobes. These features suggested lymphangitic carcinomatosis.

### 2.2 Outcome and Follow‐Up

**FIGURE 1:**
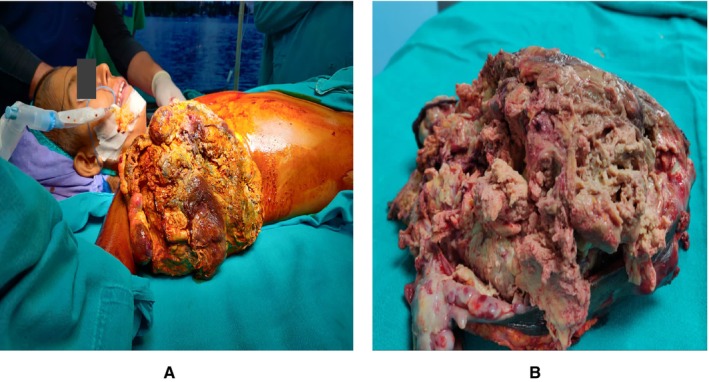
(A) Pre‐ Op Phyllodes Tumor—Fungating mass in right axilla originating from previous mastectomy bed with bleeding, purulent discharge and multiple black necrotic patches. (B) Post Op Phyllodes Tumor.

**FIGURE 2:**
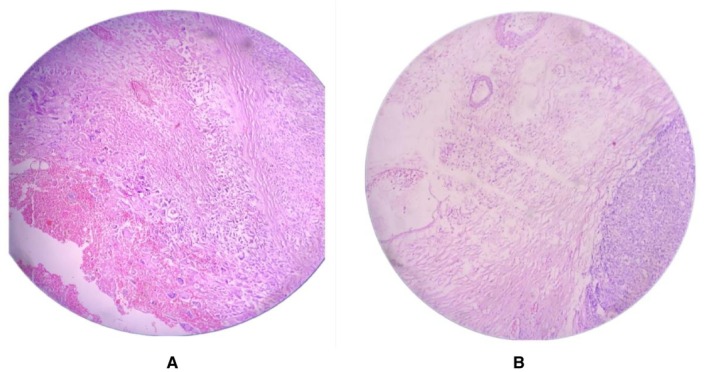
(A) Malignant spindle cell neoplasm with minimal epithelial component. The neoplastic stromal cells show pleomorphism, high N:C ratio, hyperchromatic to vesicular nuclei with abundant eosinophilic cytoplasm. (B) Microscopic Malignant Phyllodes with heterologous sarcomatoid elements.

The patient underwent wide local excision of the mass in the right axilla, which originated from a previous mastectomy scar. The specimen, measuring 7 × 12 × 16 cm, was a fungating mass with ulcerated and necrotic skin, along with foul‐smelling purulent discharge and bleeding (Figure 1). Histopathological examination confirmed a malignant phyllodes tumor (Figure 2). Immunohistochemical testing (e.g., p63, CK, CD34, Ki‐67) was not performed due to resource limitations. Hormonal profiling was not performed due to financial constraints. The patient was referred for chemotherapy and radiotherapy for further management.

## 3 Discussion

Phyllodes tumors are rare, accounting for less than 1% of all breast tumors, characterized by prominent intracanalicular architectural patterns with leaf‐like stromal fronds covered by epithelium and hypercellularity [1]. Recent studies on the molecular pathogenesis of PTs indicate that there are frequent Mediator Complex Subunit 12 (MED12) somatic mutations in both fibroadenomas and PTs, providing evidence that they share a common origin [2]. Of all phyllodes tumors, 5%–25% are malignant [5]. We present the case of an Asian woman presenting with a recurrent malignant phyllodes tumor of the breast.

A study conducted by Abe et al. reported that phyllodes tumors usually occur in middle‐aged women (35–55 years), that is, during the 4th and 5th decades of life [1], whereas our patient, aged 29, presented significantly younger than the typical range (40–45 years), with a right‐sided giant tumor. Ethnicity may play a role, as higher rates are reported in Asian women. Jayaraman et al. reported that the tumor is firm to hard with a well‐defined cut surface that shows tan‐pink to gray white color [2] whereas in our study the mass was found to be hard, painful, fungating, associated with bleeding and necrotic changes. This suggests that clinicians should consider a broad picture of the gross appearance of phyllodes tumors. Phyllodes tumors are usually well‐circumscribed and nonpainful, with an average size of 5 cm [1]. In our case, the tumor measured 7 × 10 × 16 cm with ulcerated and necrotic skin. Phyllodes tumors more commonly affect the left breast, contrary to our case in which it occurred in the upper quadrant of the right breast [3]. This difference in tumor location suggests that phyllodes tumors can occur in different locations in the breast, complicating the diagnostic approach. Although their metastatic potential is very low, they most commonly spread to the lungs, followed by the bones, heart, and liver [3]. However, in our case, there was no muscle invasion or intrathoracic extension, and the CT brain and bone scans were also normal. However, in our case, the definitive diagnosis was complicated by the presence of small lung nodules and right‐sided pleural effusion that raised concern for metastatic disease. One unique feature of giant phyllodes tumors is that they can lead to hypoglycemia irrespective of whether they are benign or malignant; however, no hypoglycemia has been described in our case [4].

The diagnosis of malignant Phyllodes tumor is challenging because of its similar clinical presentation and radiographic findings to benign lesions, making early detection of the tumor toilsome as ultrasound and mammography lack specificity for malignant Phyllodes tumor [1]. Clinicians should consider histopathological analysis as the gold standard for the diagnosis of these tumors [5]. Histologically, malignant PTs show high mitotic activity, stromal overgrowth, and heterologous elements. Diagnostic confusion with metaplastic carcinoma is possible, as both may present with spindle cells. The 2023 Curr Oncol review [9] emphasizes the importance of IHC markers such as p53, CD34, and Ki‐67, which can aid in distinguishing between histological subtypes and predicting recurrence. Our case lacked IHC due to resource constraints, limiting our ability to resolve the histological ambiguity post‐MRM.

Although surgery remains the mainstay treatment, the role of adjuvant therapy is debated. A recent meta‐analysis [10] suggests potential benefit from radiotherapy in malignant PTs, though conclusions about chemotherapy remain inconclusive. Notably, recurrence risk is tied more to tumor biology than margin width beyond 1 cm. In our case, recurrence may have stemmed from inadequate initial resection and absence of adjuvant treatment.

## 4 Conclusions

This case report highlights the diagnostic and therapeutic challenges associated with recurrent malignant phyllodes tumors, which despite their rarity, have a high recurrence rate and can mimic other aggressive breast malignancies. The overlapping histopathological features with metaplastic carcinoma complicate the diagnosis, making effective treatment strategies critical. This case emphasizes the need for increased clinical awareness and a tailored approach to manage aggressive and recurrent tumors.

## Author Contributions

**Ahmad Naeem:** validation, writing – review and editing. **Asim Ali:** conceptualization, writing – original draft, writing – review and editing. **Ali Gohar:** data curation, formal analysis, writing – original draft, writing – review and editing. **Muhammad Husnain Ahmad:** validation, visualization. **Masab Ali:** visualization, writing – original draft, writing – review and editing. **Abdul Rehman:** formal analysis, validation, writing – original draft, writing – review and editing. **Furqan Abbas Bajwa:** visualization, writing – original draft. **Muhammad Haseeb:** visualization, writing – review and editing.

## Ethics Statement

The authors have nothing to report.

## Consent

A written informed consent was obtained from the patient based on the journal's policies.

## Conflicts of Interest

The authors declare no conflicts of interest.

## Data Availability

The authors have nothing to report.
